# Radio Frequency Induction Welding of Silver Nanowire Networks for Transparent Heat Films

**DOI:** 10.3390/ma14164448

**Published:** 2021-08-08

**Authors:** Jisoo Oh, Long Wen, Hyunwoo Tak, Heeju Kim, Gyowun Kim, Jongwoo Hong, Wonjun Chang, Dongwoo Kim, Geunyoung Yeom

**Affiliations:** 1 School of Advanced Materials Science and Engineering, Sungkyunkwan University, Suwon 16419, Korea; jsoh9689@skku.edu (J.O.); moon322223@naver.com (L.W.); stampede524@gmail.com (H.T.); vosejrhd1@naver.com (H.K.); kgw0401@nate.com (G.K.); hong9794799@naver.com (J.H.); wonjun082003@g.skku.edu (W.C.); dwkim111@gmail.com (D.K.); 2 SKKU Advanced Institute of Nano Technology (SAINT), Sungkyunkwan University, Suwon 16419, Korea

**Keywords:** silver nanowires, nano welding, induction heating, rf frequency, transparent heat film

## Abstract

Transparent heat films (THFs) are attracting increasing attention for their usefulness in various applications, such as vehicle windows, outdoor displays, and biosensors. In this study, the effects of induction power and radio frequency on the welding characteristics of silver nanowires (Ag NWs) and Ag NW-based THFs were investigated. The results showed that higher induction frequency and higher power increased the welding of the Ag NWs through the nano-welding at the junctions of the Ag NWs, which produced lower sheet resistance, and improved the adhesion of the Ag NWs. Using the inductive welding condition of 800 kHz and 6 kW for 60 s, 100 ohm/sq of Ag NW thin film with 95% transmittance at 550 nm after induction heating could be decreased to 56.13 ohm/sq, without decreasing the optical transmittance. In addition, induction welding of the Ag NW-based THFs improved haziness, increased bending resistance, enabled higher operating temperature at a given voltage, and improved stability.

## 1. Introduction

Transparent and flexible heaters are visually transparent devices that contain an electrically conductive layer. When a current across the transparent heater generates heat by the Joule effect, this heat can be efficiently used in many devices. Consequently, numerous applications require transparent and flexible heaters, and the associated market comprising many types of printed electronic devices such as smart windows, defoggers, displays, sensors, etc., is growing fast [1,2,3,4,5]. Great attention has been devoted to various flexible and transparent heaters (or electrodes) based on printed electronics due to different reasons such as devices having nonplanar and flexible substrates, the potential scarcity of indium, and the lack of transparency in the near-infrared spectrum [6,7,8]. In these printed electronic products, conductive materials with low resistance, high transmittance, high flexibility, etc., are essential in the performance of the devices, and various conductive materials such as transparent conductive oxide, metallic nanowires, conductive polymers, and carbon-based electrodes have been investigated [9,10,11,12,13,14].

Among the various conductive nanomaterials investigated for printed electronic products, silver nanowires (Ag NWs) are considered to be one of the ideal alternative candidates for future applications in electronics, owing to their advantageous characteristics, which include their ductility, low sheet resistance, and low cost. Moreover, they can be fabricated by facile low-cost solution-based processes, such as spin coating, spray coating, soft imprinting, and vacuum filtration, and electrospinning process coating [15,16,17,18,19,20,21,22]. However, there are several issues to be solved for the practical application of Ag NWs in the area of electronics, such as the significant contact resistance of the nanowire junctions due to the weak bonding between nanowires, high surface roughness, and instability due to the high specific surface area. All the factors mentioned above can degrade the performance of Ag NW networks and impede the application of Ag NW networks in electronics [23,24,25,26].

To improve the electrical and mechanical characteristics of the transparent electrode using Ag NWs, the welding process is necessary to enhance their performance. Ag NW welding using various methods has been investigated, such as heating, plasmonic effect, capillary force, chemical treatment, laser irradiation, and radiofrequency [27,28,29,30,31,32,33,34,35,36]. The welding treatment can significantly reduce the contact resistance of the Ag NWs. Among these methods, induction welding using radiofrequency has additional advantages, because it can heat and weld only the junction area through the eddy current induced on the Ag NWs by the electromagnetic field generated by rf power, and it is a self-terminating process, where when the welding process is complete, the welding stops itself [37,38,39,40,41].

In this paper, we use induction heating systems operated at various radio frequencies to investigate the nanowire network’s welding characteristics according to the radio frequency and power for the Ag NWs with different resistivities, and explore the effects of Ag NW induction welding on the characteristics of Ag NW-based transparent heat films (THFs).

## 2. Experimental

**Figure 1:**
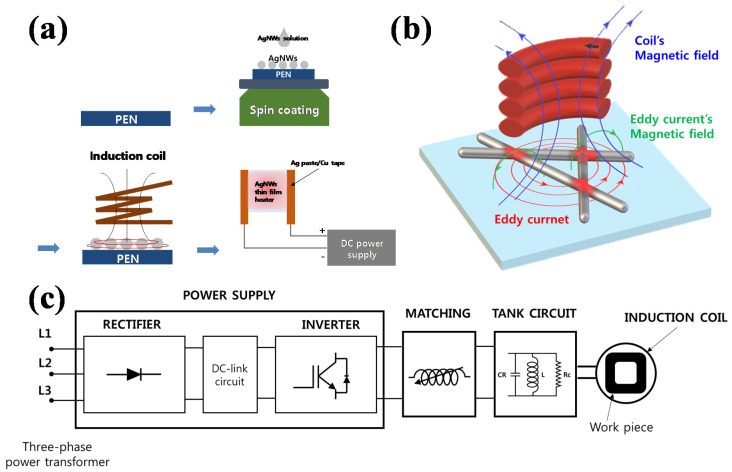
Schematics of (**a**) the processes forming Ag NWs thin film heaters, and (**b**) the principle of induction heating on the surface of Ag NW networks. (**c**) Schematic of the basic induction heating system.

In this experiment, commercially available Ag NWs (NANOPYXIS, of 22 ± 5 µm length, and 25 ± 3 nm diameter) were used. These Ag NWs were diluted with isopropyl alcohol (IPA) to form a 0.05 wt.% Ag NW solution. A polyethylene naphthalate (PEN) substrate was coated with the Ag NW solution. Before coating with the Ag NW solution, the substrates were cleaned in sequence using alcohol, and deionized water (DIW) by sonication. The Ag NW film was coated by spin coating of the Ag NW solution at 500 rpm for 5 s, and 4000 rpm for 60 s. Uniform nanowire films with the initial sheet resistances of 30, 50, 100, and 300 ohm/sq could be obtained on the PEN through the multiple coating. To demonstrate the applications of these Ag NW coated transparent flexible films, 25 mm × 25 mm THFs were fabricated using three Ag NWs films (a non-welded Ag NWs film and Ag NW films welded by 100 and 800 kHz inductive heating) for 60 s at 6 kW. The THFs films were fabricated by attaching electrodes on the sides of the films with silver paste and copper tape. The silver paste was painted at the edges of the heat film, and the copper tape was used to connect the heat film with outside electrical wires. Figure 1a shows the process sequence of the THF fabrication by Ag NW coating on the PEN.

The Ag NWs coated substrates were welded by inductive heating. Figure 1b,c show the schematic diagram of the induction welding principle and basic induction heating system, respectively. The induction heating system used in this study is commercially available equipment (Lihua Technology Industrial Co. Ltd., Guangdong, China). The applied input power of the induction heater can be controlled and set to a maximum of 6 kW, and the frequency was fixed at 100, 450, and 800 kHz for each induction heating system. For all systems, the distance between the inductive coil and the substrates was maintained at 5 mm. For the welding, the inductive power of 1–6 kW and the welding time of 30–600 s were varied to observe the resistance change of Ag NWs film. 

To improve the stability of the Ag NW-based THF during the heating experiment, the Ag NW networks on the PEN substrates were plasma-treated with C_4_F_8_ plasma to cover the Ag NW network with a polymer layer. To deposit the fluorocarbon polymer layer on the Ag NW networks, the Ag NW-coated THFs were exposed to c-C_4_F_8_ (octa-fluorocyclobutane-fluorocarbon molecules with a circular structure) plasma for 30 s at the substrate temperature of 20 °C. The plasma was operated with a 60 MHz capacitively coupled plasma system, at a constant power of 150 W, and at the operating pressure of 30 mTorr C_4_F_8_ [42].

To order to observe the changes of the Ag NWs morphologies, samples were prepared by coating Ag NW on 25 mm × 25 mm PEN using the spin coating method as mentioned above. The welding characteristics of the Ag NWs were observed using field emission scanning electron microscopy (FE-SEM; S-4700 Hitachi). The changes of the Ag NWs morphology were observed using atomic force microscopy (AFM; INOVA, MA, USA). Optical transmittance was observed using optical microscopy and measured using ultraviolet–visible (UV–Vis) spectroscopy (UV-3600, Shimadzu, Kyoto, Japan). The haze values were measured by haze meter (BYK-Gardner haze-gard i). The sheet resistance was measured using a four-point probe (CMT-SR2000N, AiT). The mechanical integrity of the Ag NWs film (50 mm × 20 mm PEN) was measured using a lab-made bending test system bent to a radius of curvature of 5 mm. The temperature of the THFs was measured as a function of input voltage and heating/cooling cycle times using a thermocouple that directly touches the surface of THFs, and a thermographic camera (FLIR).

## 3. Results and Discussion

Figure 1b shows that when an alternating electrical current is applied across a conductive coil, an alternating magnetic field with the same frequency is generated, and according to the Faraday effect, if a conductor is located within the alternating magnetic field, an alternating electric current (eddy current) will be induced with the same frequency as the magnetic field. The eddy currents heat the material according to the well-known Joule effect [40,43]. In our experiment, when eddy current flows to the Ag NW network, a hot spot is generated at the junctions due to the high resistance at the junction, and the junctions are preferentially melted without heating the remainder of the Ag NW area. The heating rate of the Ag NW network is dependent on the operating frequency of induction power because, according to Faraday’s law of induction, the induced current is correlated with the operating frequency. The ability to handle internally applied and localized heating with high heat rates prevents significant thermal stress, degradation, or deformation after the welding [43,44,45,46]. 

The operating frequency applied for radio frequency induction heating is usually higher than 20 kHz and in less than 1 MHz, depending on the application. And at frequencies below 100 kHz, the skin depth is large, which makes it inefficient to apply to nanomaterials [37,47]. Therefore, three frequency systems were selected within that frequency range to investigate the effect of induced power and frequency on the change in sheet resistance of the Ag NW network. The welding process was performed for 60 s while changing the power from 1 to 6 kW. 

**Figure 2:**
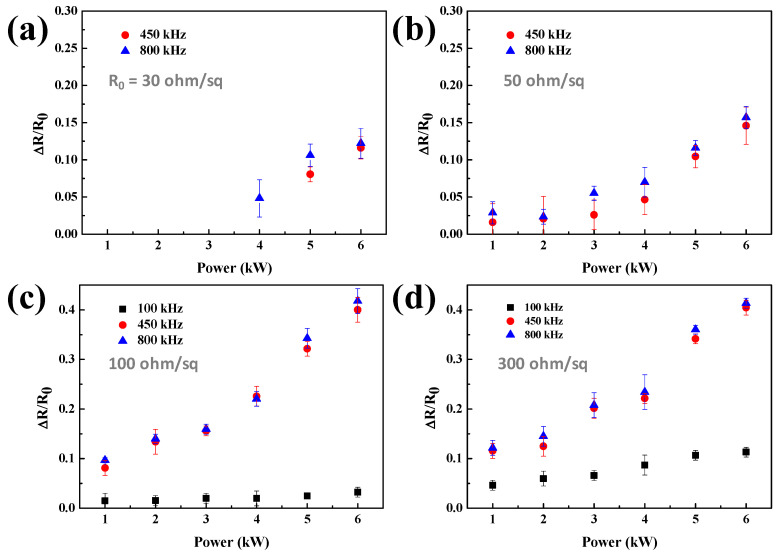
Change of Ag NWs sheet resistance by the welding with different frequencies of 100, 450, and 800 kHz and with 1–6 kW power for 60 s for the Ag NW networks with the initial sheet resistances of (**a**) 30, (**b**) 50, (**c**) 100, and (**d**) 300 ohm/sq.

Figure 2 shows the results for the initial sheet resistance of (a) 30, (b) 50, (c) 100, and (d) 300 ohm/sq. The figure also shows that the higher the initial resistance, the more significant resistance change that was observed during nanowire welding using induction heating. Figure 2a shows that for the Ag NW network with 30 ohm/sq, no change in resistance was observed, even when 6 kW of power was applied at 100 kHz, while the resistance changes of ~11.6 and ~12.2% were observed for 450 and 800 kHz, respectively, at 6 kW. Figure 2b–d show that as the initial resistance increased to 50, 100, and 300 ohm/sq, respectively, the sheet resistance reduction percentage (ΔR/R_0_) at a given power was further increased by showing 8.1% at 450 kHz and 9.6% at 800 kHz for 1 kW of power, and 40% and 43.7% at 450 and 800 kHz, respectively, for 6 kW power. In addition, even though the reduction percentage is small, the reduction of sheet resistance was also observed even with 100 kHz for 100 and 300 ohm/sq of Ag NW sheet resistance. 

**Figure 3:**
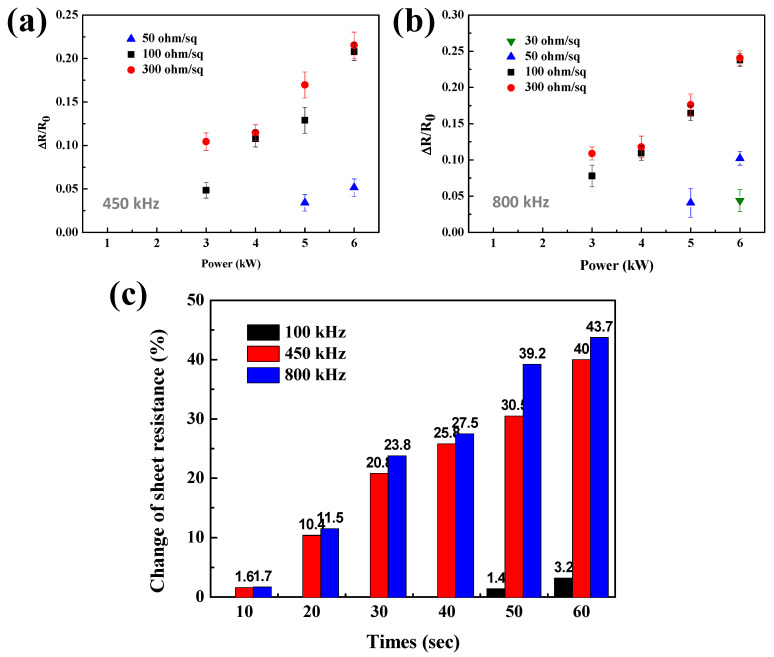
Sheet resistance change according to the inductive heating of the Ag NW network for 30 s at (**a**) 450 and (**b**) 800 kHz. (**c**) Comparison of sheet resistance change over induction heating time with 6 kW power applied to each frequency for 100 ohm/sq of initial resistance Ag NW-coated thin films.

Figure 2c,d show that the differences in sheet resistance change of Ag NW networks between 450 and 800 kHz are not significant, even though they are significantly different from those welded at 100 kHz. Therefore, to confirm the difference between the two frequencies, the induction heating for 30 s with the power of 1–6 kW using 450 and 800 kHz was also investigated, and Figure 3a,b show the respective results. As shown in Figure 3a, even when 6 kW was applied at the frequency of 450 kHz, there was no change of resistance in the 30 ohm/sq Ag NW network. However, the resistance change could be observed for the Ag NW network with the initial resistance of 50 ohm/sq or higher. When 6 kW power was applied for 30 s, the resistance change was ~5% for the 50 ohm/sq Ag NW network, ~20.8% for the 100 ohm/sq Ag NW network, and ~21.5% for the 300 ohm/sq Ag NW network. In contrast, Figure 3b shows that at 800 kHz frequency and 6 kW, the resistance change of ~4.4% was observed for the initial resistance of 30 ohm/sq Ag NW networks, and the sheet resistance change of ~23.8 and ~24.1% for the 100 and 300 ohm/sq, respectively, could be observed. Figure 3c compares the changes in sheet resistance of 100 ohm/sq Ag NW networks as a function of welding time from 10 to 60 s at 6 kW for different frequencies. Although after the welding, a change in sheet resistance is seen at all frequencies, the effect is negligible at 100 kHz, and the rate of resistance change increased with increasing frequency. 

**Figure 4:**
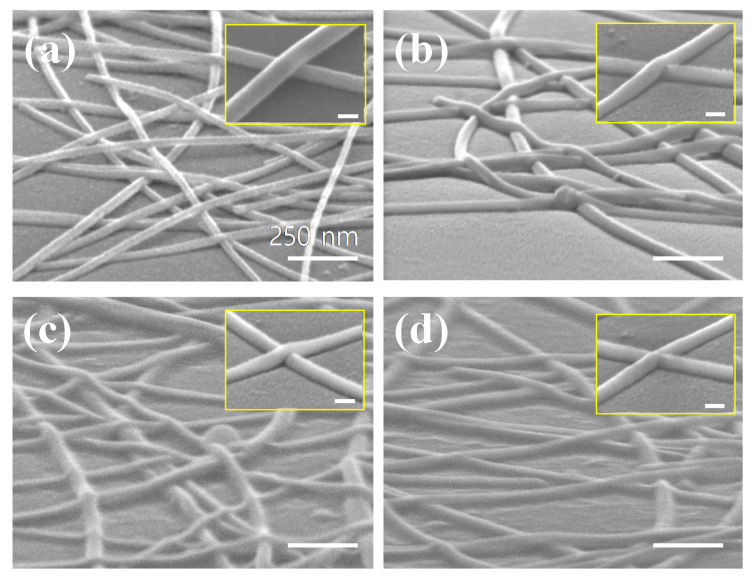
SEM image of the 100 ohm/sq Ag NW networks after inductive welding. (**a**) After the coating on the PEN substrate without welding; (**b**) after welding at 100 kHz; (**c**) after welding at 450 kHz; and (**d**) after welding at 800 kHz at 6 kW for 60 s. All of inset images are the morphology of nanowire junctions (scale bar: 30 nm).

Figure 4 shows the results of the morphologies of the Ag NW networks on PEN substrates before and after the welding for 60 s at 6 kW at different frequencies that were observed by SEM for the Ag NW network of 100 ohm/sq. Figure 4a shows the Ag NW networks after the coating on the PEN substrate without welding, (b) shows the Ag NW networks after welding at 100 kHz, (c) shows after welding at 450 kHz, and (d) shows after welding at 800 kHz. Figure 4a shows that after the coating without welding, the Ag NW junctions were not welded, and were separated from each other. 

However, Figure 4b–d show that with increasing the operating induction frequency, increased welding of the Ag NW junction parts could be observed, which after the welding process resulted in the decrease of sheet resistance.

In particular, Figure 4d shows that for the welding at 800 kHz, the Ag NW junction showed complete welding, without changing the morphology of the nanowire. However, Figure 4d inset image shows that even though most of the Ag NW junctions were welded completely at 800 kHz, some of the Ag NW junctions having stacked multiple junctions appeared to not be completely welded. This indicates possible difficulty in welding the stacked Ag NW junctions; therefore, difficulty in reducing the sheet resistance by induction welding for low sheet resistance Ag NW networks containing more stacked Ag NW junctions, such as 30 ohm/sq, was observed in Figure 2 and Figure 3.

**Figure 5:**
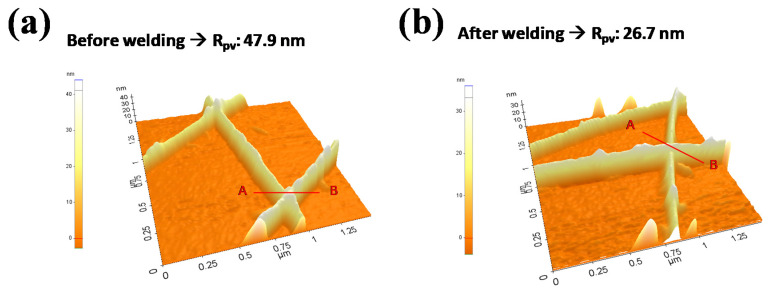
Surface roughness of a single Ag NW junction measured by 2-dimensional AFM. (**a**) before and (**b**) after welding at the 800 kHz frequency with the condition shown in Figure 4d.

The surface roughness of a single Ag NW junction before and after the welding at the 800 kHz inductive heating condition shown in Figure 4d was investigated using AFM. Figure 5a,b show the results for the Ag NWs before and after the welding, respectively, and in Figure S1 for line scan data of the single Ag NW and the Ag NW junction before and after the welding (detailed height changes for single Ag NW and before/after welding Ag NW can be found in Figure S1, Supporting Information). Before the welding, the height of the Ag NW junction was ~47.9 nm, while the height of the single Ag NW was 25 ± 3 nm. 

Therefore, before the welding, a single Ag NW was physically resting on top of another single Ag NW at the junction. After the welding, the height of the Ag NW junction was decreased to ~26.7 nm, indicating the almost complete fusion of the two Ag NWs at the junction, as shown in Figure 4d. In addition, after the welding, the surface roughness of the Ag NWs network was significantly decreased, due to the fusion of the two Ag NWs at the junction. 

**Figure 6:**
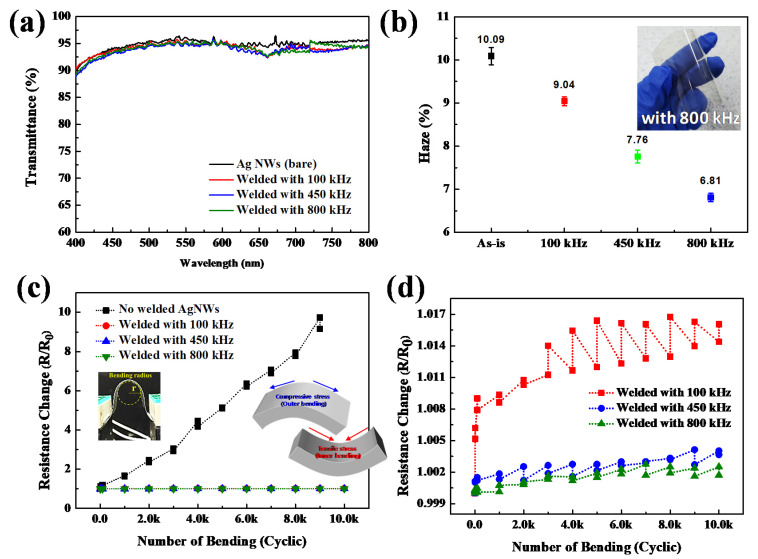
(**a**) Optical transmittance, (**b**) haze, and (**c**,**d**) resistance change with bending cycles of the Ag NW networks on PEN that were not, and were welded at 100, 450, and 800 kHz frequency at 6 kW for 60 s.

Various physical properties of the Ag NW networks coated on PEN after the welding were investigated. Figure 6a shows the optical transmittance before and after the welding of Ag NW networks on PEN using different induction frequencies at 6 kW for 60 s. The Ag NW network with the sheet resistance of 100 ohm/sq before welding was used. Figure 6a shows that before welding, the optical transmittance of the Ag NW network with 100 ohm/sq was 96.1% at 550 nm wavelength. 

After welding, the optical transmittances of the Ag NW networks welded at 100, 450, and 800 kHz were 95.5%, 95.0%, and 95.1%, respectively, indicating that after welding, there were no noticeable changes in optical transmittance for all frequencies. Figure 6b shows the haze percentage of the Ag NW networks in Figure 6a. Before the welding, the haze percentage was 10.09%, and, after the welding at 100, 450, and 800 kHz, the haze percentage was continuously decreased to 9.04%, 7.76%, and 6.81%, respectively, with increasing the induction frequency. This decrease in haze is due to the decreased light scattering and lower angle scattering by the increased fusion of Ag NW junctions [29]. 

Mechanical strength was also measured by cyclic bending test of the Ag NW networks coated on PEN before and after welding at different induction frequencies with the conditions in Figure 6a, and Figure 6c,d shows the changes in sheet resistance measured as a function of the bending cycle. For the bending test, the Ag NW network with initial resistance of about 100 ohm/sq was spin-coated on a 50 × 20 mm PEN substrate. The bending test was performed by repeating tensile/compressive cycles up to 10,000 cycles with a radius of 5 mm using a bending machine. Figure 6c shows that the Ag NW network coated on PEN without welding showed rapid increase of sheet resistance, and before the cyclic bending cycle reached 10,000 cycles, the Ag NW network was broken by showing infinite resistivity. In contrast, after welding, the Ag NW networks appeared to show negligible change in resistance with increasing bending cycles, regardless of the induction frequency. However, Figure 6d shows that, when the change of resistance was more accurately observed, the higher induction frequency showed lower change in sheet resistance by showing the resistance change of 1.62%, 0.04%, and 0.02% for 100, 450, and 800 kHz condition, respectively, after 10,000 cycles. The increase in sheet resistance can be attributed to broken junctions in the random Ag NW network. The more changes in sheet resistance under a tensile stress state than a compressive stress state indicates that the random Ag NW network is more vulnerable to the failure under a tensile stress. It is believed that non-linear behavior of resistance change observed at 100 kHz is related to more significant resistance changes due to less welded junctions. It is also believed that the smaller change of sheet resistance for the higher induction frequency is related to the increasing number of fully welded Ag NW junctions.

**Figure 7:**
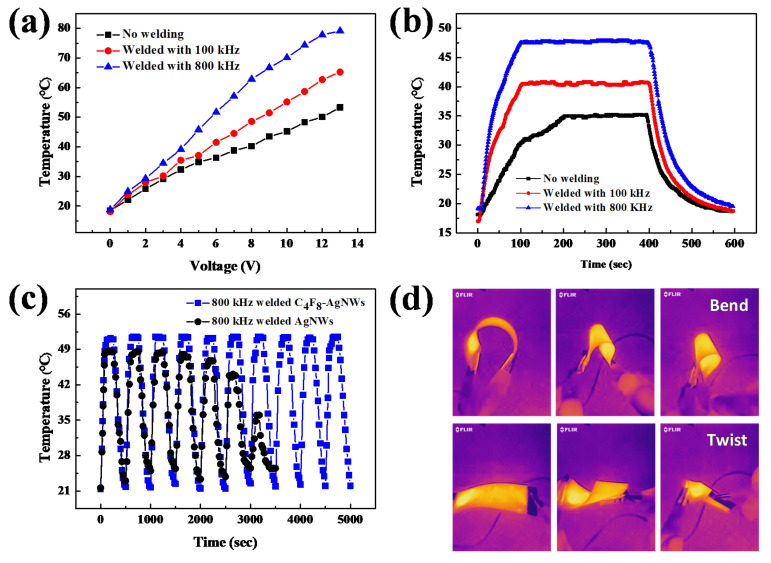
Ag NW THFs before and after welding at 100 and 800 kHz at 6 kW for 60 s. (**a**) Temperature of the THFs as a function of applied voltage. (**b**) Time-dependent temperature profile of the THFs at 6 V of applied voltage. (**c**) Temperature response of the THFs (welded at 800 kHz at 6 kW for 60 s) over 10 switching cycles with the applied voltage of 6 V (one cycle = 250 s on-time/250 s off-time) with, and without, a passivation layer formed by a C_4_F_8_ plasma. (**d**) Thermal imagery of the Ag NW THFs welded at 800 kHz with the condition shown in (**a**). For (**c**,**d**), to protect the Ag NW network from the environment during the long-time exposure to atmosphere, the THFs were coated with a fluorocarbon polymer layer formed by a C_4_F_8_ plasma.

Using the Ag NWs coated PEN, THFs were fabricated, and their heater characteristics were investigated for the Ag NW networks before and after the welding at 100 and 800 kHz at 6 kW for 60 s, and Figure 7 shows the results. The sheet resistivity of the Ag NW network before the welding was ~100 ohm/sq, while after the welding at 100 and 800 kHz, the resistivities were decreased to ~90.6 and ~56.1 ohm/sq, respectively, without changing the optical transmittance. The temperature of the THFs was measured using a thermocouple attached to the surface of the THFs. Figure 7a shows the saturated temperature of the Ag NW-based THFs measured as a function of applied voltage. 

Figure 7a shows that the temperature for the non-welded Ag NW-based THF was ~36 °C at 6 V, and increased to 53 °C at 13 V. In the case of Ag NW-based THF welded at 100 and 800 kHz, the temperature was increased to ~65 and ~79 °C at 13 V, respectively. Therefore, the increase of applied voltage increased the saturated temperature of all THFs; however, at the same voltage, the highest temperature was observed for the Ag NW network welded at 800 kHz, and the lowest temperature for the Ag NW network before welding. Figure 7b shows the temperature response over time for the Ag NW-based THFs at 6 V DC bias voltage with the Ag NW networks before and after the welding at 100 and 800 kHz. For non-welded Ag NW-based THF, it reached the saturation temperature of ~36 °C at ~200 s, while the THFs welded at 100 and 800 kHz reached the saturation temperature of ~41 and ~48 °C, respectively, at ~100 s. In addition, the THFs with the welded Ag NW networks showed more stable temperature increase rate, compared to that with the non-welded Ag NW network.

Figure 7c shows the result of the cyclic switching test using the Ag NW-based THFs welded at 800 kHz. The test was conducted at the operating voltage of 6 V. Cyclic heating and cooling tests were performed for 10 cycles (1 cycle = 250 s on-time/250 s off-time). If Ag NWs are exposed to atmospheric environment at high temperature for a long time, due to the inherent properties of nanowires, the Ag NWs can be easily damaged by the external environment, such as by oxidation. Therefore, before the cyclic switching test that requires a lengthy exposure to air at high temperature, the Ag NW-based THF surfaces were exposed to a C_4_F_8_ plasma to form a fluorocarbon passivation layer with the condition in the experimental section, and the Ag NW-based THFs with and without the passivation layer were also compared. Figure 7c shows that when the Ag NW-based THF was not passivated, the THFs were gradually degraded by oxidation during the cyclic switching test; however, when the passivated Ag NW-based THF was repeatedly heated to ~53 °C, and cooled to near room temperature, it showed good response time, operational stability, and repeatability (the passivated Ag NW-based THF showed slightly higher saturation temperature at the same operating voltage than that without the passivation layer, but the optical transmission and the temperature behavior were similar to those without passivation). Therefore, for the passivated Ag NW-based THF, stable cyclic temperature behavior was observed during the switching test. Figure 7d shows the optical image of the passivated Ag NW-based THF fabricated on PEN and the images of the heat radiation measured with an infrared (IR) camera. The IR camera was used to visually confirm that the welded Ag NW-based THFs not only showed higher temperature rise compared to the unwelded Ag NW-based THFs, but also showed more uniform temperature rises over the entire area of the heat film, even when bent and twisted (a more detailed figure is shown in Figure S2 of the supporting information).

## 4. Conclusions

In this work, Ag NW networks on PEN with different sheet resistances were welded by induction heating with different induction frequencies and power, and the optical, electrical, and mechanical properties of the Ag NW coated PEN before and after the welding was investigated. The eddy current induced by induction heating welds only the Ag NW junction area and remotely from the junction area; therefore, it was an efficient Ag NW welding method showing no change in optical transmittance after the welding. The results showed that the higher the induction frequency and higher the induction power, the more significant the welding effect that could be observed for the Ag NW network with the same resistivity. For the same induction frequency and power, the Ag NW network with higher sheet resistance showed higher welding effect, by showing more significant change in resistivity after welding. The induction welding of the Ag NW networks at higher frequency not only decreased the sheet resistance, but also decreased the haze by decreasing the total height of the Ag NWs during the joining of the junction area, without changing the optical transmittance. The Ag NW network on PEN welded at higher induction frequency also showed better stability during the cyclic bending test, and THF fabricated with welded Ag NW networks by induction heating at the higher frequency showed higher temperature rise at a given applied voltage. It is believed that the inductive welding of nanowire films would be widely applicable to THF as well as the various flexible electronics such as solar cells, touch panels, and biosensors.

## Data Availability

Not applicable.

## Supplementary Materials

The following are available online at https://www.mdpi.com/article/10.3390/ma14164448/s1, Figure S1: Line can data of the single Ag NW and the Ag NW junction before and after the welding. Figure S2: IR images of Ag NW THFs while bending and twisting at 6 V. (induction heating welded at 800 kHz condition). To protect Ag NW network from the environment, the THFs were coated with a fluorocarbon polymer layer.
