# Supplementary material for: Radio Frequency Induction Welding of Silver Nanowire Networks for Transparent Heat Films
Source: Materials (Basel). 2021 Aug 8;14(16):4448. doi: 10.3390/ma14164448 (PMC8400299; doi:10.3390/ma14164448)
Supplement: Supplementary file 1 [file materials-14-04448-s001.zip › materials-1307573.pdf]

materials

MDPI

Supplementary Materials

Radio Frequency Induction Welding of Silver Nanowire Networks for Transparent Heat Films

Jisoo Oh [1,†], Long Wen [1,†], Hyunwoo Tak [1], Heeju Kim [1], Gyowun Kim [1], Jongwoo Hong [1], Wonjun Chang [1], Dongwoo Kim [1] and Geunyoung Yeom

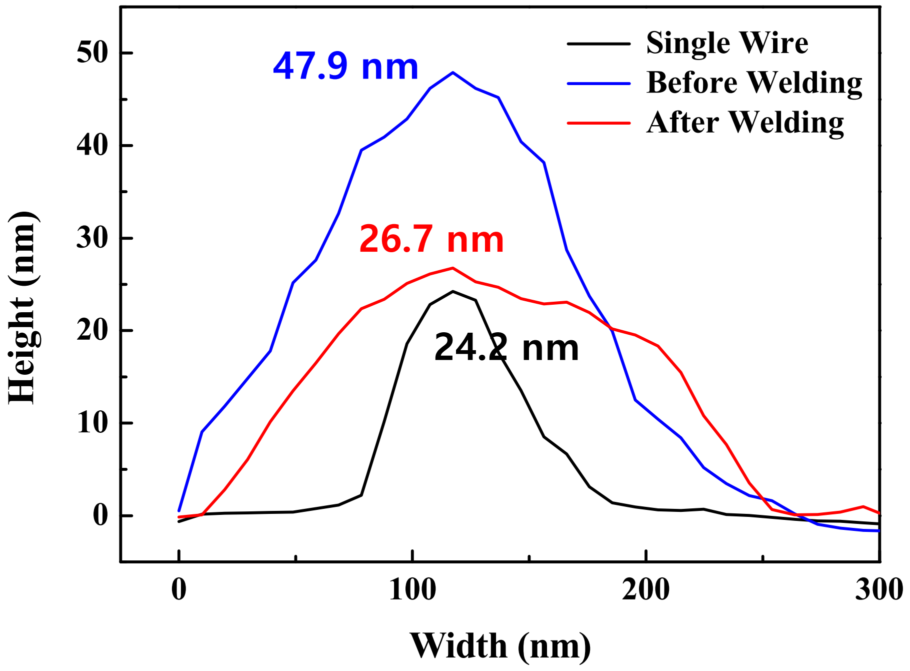

Figure S1. Line can data of the single Ag NW and the Ag NW junction before and after the welding.

As shown in Figure S1, before the welding, the height of the Ag NW junction was ~47.9 nm, while the height of the single Ag NW was ~24.2 nm. Therefore, before the welding, a single Ag NW was physically resting on the top of another single Ag NW at the Ag NW junction. However, after the welding, the height of the Ag NW junction decreased to ~26.7 nm, indicating almost complete fusing of two Ag NWs at the junction, as shown in Figure 4(d).

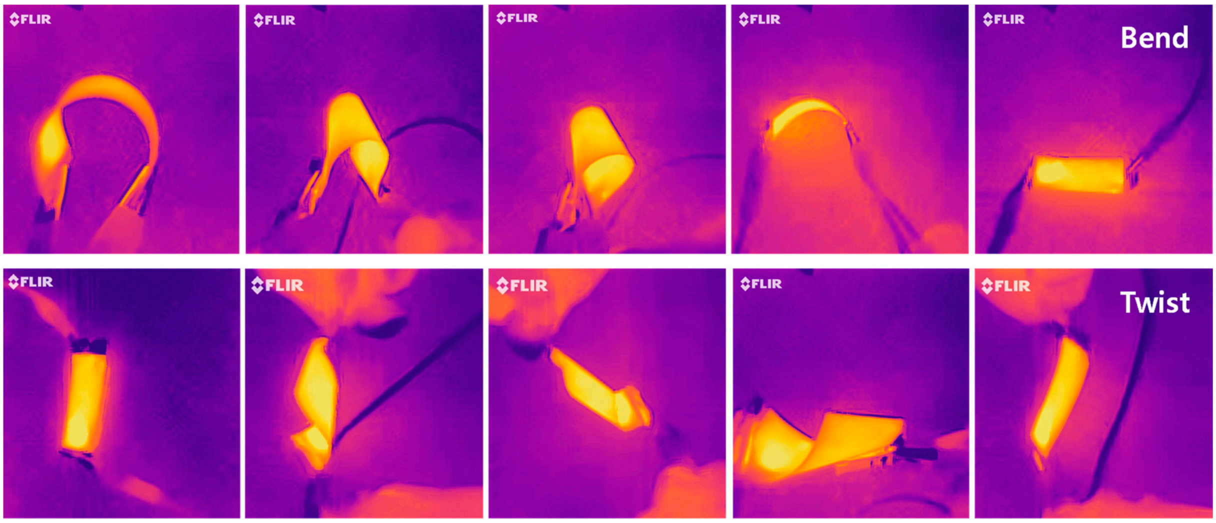

Figure S2. IR images of Ag NW THFs while bending and twisting at 6 V. (induction heating welded at 800 kHz condition). To protect Ag NW network from the environment, the THFs were coated with a fluorocarbon polymer layer.

Figure S2 show the optical images of the passivated Ag NW-based THF fabricated on PEN and the images of the heat radiation measured with an infrared (IR) camera. Using the IR camera, it could be visually confirmed that the welded Ag NW-based THFs showed not only higher temperature rise compared to the unwelded Ag NW-based THFs, but also a more uniform temperature rise over the entire area of the heat film even when bent and twisted.
